# Novel Carboxylated PANI/MWCNT Dispersions and Impregnated Cellulose Substrates for Photocatalytic Methylene Blue Dye Removal

**DOI:** 10.3390/nano16120735

**Published:** 2026-06-13

**Authors:** Silvia Dimova, Katerina Zaharieva, Petar D. Petrov, Maria Shipochka, Rositsa Titorenkova, Petya Todorova, Ognian Dimitrov, Denitsa Nicheva, Hristo Penchev

**Affiliations:** 1 Institute of Polymers, Bulgarian Academy of Sciences, Akad. G. Bonchev St., Block 103A, 1113 Sofia, Bulgaria; ppetrov@polymer.bas.bg; 2 Institute of Mineralogy and Crystallography “Acad. I. Kostov”, Bulgarian Academy of Sciences, Acad. G. Bonchev St., Block 107, 1113 Sofia, Bulgaria; rositsatitorenkova@imc.bas.bg (R.T.); vepege@abv.bg (P.T.); 3 Institute of General and Inorganic Chemistry, Bulgarian Academy of Sciences, Acad. G. Bonchev St., Block 11, 1113 Sofia, Bulgaria; shipochka@svr.igic.bas.bg; 4 Institute of Electrochemistry and Energy Systems “Acad. Evgeni Budevski”, Bulgarian Academy of Sciences, Acad. G. Bonchev St., Block 10, 1113 Sofia, Bulgaria; ognian.dimitrov@iees.bas.bg (O.D.); d.vladimirova@iees.bas.bg (D.N.)

**Keywords:** carbon nanotubes, polyaniline, hybrid materials, photocatalytic ability, methylene blue, dye

## Abstract

Hybrid conductive materials have attracted increasing attention due to their combined electrical conductivity, mechanical flexibility, and sustainability. In this work, new hybrid materials based on polyaniline (PANI)-wrapped multi-walled carbon nanotubes (MWCNTs) and microfibrous cellulosic substrates were developed and assessed for photocatalytic degradation of a model dye pollutant. First, in situ oxidative polymerization of aniline in formic acid (FA) was conducted in the presence of MWCNTs to afford stable dispersions of carboxylated polyaniline-wrapped carbon nanotubes (c-PANI/MWCNTs). Next, the dispersions were used for affordable impregnation of microfibrous cellulosic filter paper. The influence of the initiator type—potassium peroxodisulfate (KPS) and hydrogen peroxide—on polymer–nanotube interactions, stabilization and surface deposition was emphasized. The structural, surface, morphological and thermal properties of the obtained dispersions and cellulose nanocomposites were systematically investigated using Fourier-transform infrared spectroscopy, X-ray photoelectron spectroscopy, Raman spectroscopy, scanning electron microscopy, energy-dispersive X-ray spectroscopy and thermal gravimetric analysis. The results revealed strong interfacial interactions between c-PANI and the pristine MWCNTs, resulting in improved dispersion stability and effective and even surface deposition of the conductive c-PANI/MWCNT hybrids into the cellulose fiber mesh. The photocatalytic degradation of 5 ppm methylene blue (MB) dye in the presence of the developed nanocomposite materials under UV-A illumination was studied. The results showed that the c-PANI@MWCNT-impregnated cellulose substrates exhibited enhanced photocatalytic ability (up to 83% degree of degradation of MB dye) in comparison with the pure c-PANI.

## 1. Introduction

Conductive polymer-based nanocomposites have attracted significant attention for applications in flexible electronics, sensors, energy storage devices, and wearable technologies due to their low density, mechanical compliance, and tunable electrical properties [1,2]. Among conducting polymers, polyaniline (PANI) is attractive for practical use because of its facile synthesis, environmental stability, low cost, and reversible control of electrical conductivity through mineral or organic acid protonation doping [3]. Despite these advantages, the application of pristine PANI is restricted by its intrinsic brittleness, limited mechanical strength, and poor processability [3,4]. M. Trchová et al. reported that the synthesis of polyaniline at a stoichiometric peroxydisulfate-to-aniline mole ratio in solutions of formic acid resulted in partial ring carboxylation of polyaniline at high acid concentrations [5].

Carbon nanotubes (CNTs), owing to their exceptional electrical conductivity, high aspect ratio, and superior mechanical properties, are widely employed as nanofillers to enhance the performance of conducting polymers [6]. Incorporation of CNTs into PANI matrices leads to synergistic improvements in electrical conductivity through the formation of percolation networks and strong π–π interactions between the conjugated PANI chains and CNT surfaces. These interactions facilitate efficient charge transport while simultaneously reinforcing the polymer matrix [7,8]. In the literature, an electrochemical sensor based on an amine-functionalized Zr(IV) metal–organic framework and MWCN composite has been reported, exhibiting good sensitivity toward Cd^2+^ [9]. A biomimetic enzyme electrochemical biosensor based on tetraphenyl metalloporphyrin-functionalized multi-walled carbon nanotubes for detection of antioxidants such as tert-butylhydroquinone in plant oil has been investigated by Zou Bin et al. [10]. Xiangbo Fan et al. studied polyacrylic acid-grafted lignosulfonate/carboxymethyl cellulose and biochar composites prepared by free radical polymerization, which demonstrated good adsorption performance on Pb^2+^ and methylene blue dye [11].

Cellulose, the most abundant renewable biopolymer, possesses biodegradability, flexibility, and a porous microfibrillar architecture that is well suited for impregnation with functional nanomaterials [12]. Integration of conductive PANI/CNT hybrids into cellulose substrates represents a promising strategy for developing lightweight, flexible, and sustainable conductive materials [13]. However, achieving stable, homogeneous conductive nanodispersions that can be efficiently incorporated into a cellulose matrix while preserving good electrical performance remains a considerable challenge [14].

Different methods have been reported for the preparation of PANI/CNT hybrid materials, such as in situ polymerization, electrochemical polymerization, interfacial polymerization, solution mixing, electrophoretic methods, the electrospinning technique [15,16,17,18,19,20,21,22] and the solid-state synthesis method [23,24].

The present study describes the formation of stable PANI/CNT hybrid dispersions in formic acid and their use for direct impregnation of cellulose substrates. Beneficially, the stable PANI/CNT dispersions allow for controlled nanoscale organization of conductive networks, which is critical for achieving uniform electrical properties in cellulose-based nanocomposites. Notably, formic acid serves both as a polymerization medium for aniline and a dispersion medium for the formed PANI-modified CNT hybrids. This strategy enables enhanced CNT stabilization, improved interfacial interactions, and efficient formation of conductive networks within the cellulose matrix upon impregnation. Moreover, the method provides a scalable and environmentally benign route toward sustainable conductive composites [25].

Various organic contaminants such as dyes released from industry are harmful to human beings. Many methods have been used in recent years to solve the problems with water pollution, such as filtration, solvent extraction, chemical oxidation, photocatalytic degradation, adsorption and flotation. Among these techniques, photocatalysis has attracted considerable attention because of its environmentally friendly properties and cost-effectiveness [26,27,28,29]. Yuan et al. established that hydrothermally synthesized PANI-CNT/TiO_2_ photocatalysts degraded diethyl phthalate more efficiently than photocatalysts of the same composition obtained by sol–gel hydrolysis [30]. Qurtulen et al. investigated the photocatalytic ability of a CQDs/PANI (5 wt.%) nanocomposite for the degradation of Brilliant Green dye under visible-light irradiation. The prepared photocatalyst showed nearly 100% dye degradation [31]. Chatterjee et al. obtained SWCNT-PANI nanocomposite photocatalysts for degradation of methyl orange and rose bengal dyes under visible light [32]. Ag/AgCl–polyaniline materials have been investigated as photocatalysts for degradation of methylene blue dye under solar light [33]. PANI prepared using 2.0 M HCl and a polymerization temperature of 25 °C demonstrated maximum photocatalytic efficiency for Rh B dye removal [34]. Synthesized fibrous cellulosic substrates impregnated with meta-polybenzimidazole (PBI)-stabilized carbon nanotubes/ZnO composites have been investigated as photocatalysts for degradation of methylene blue dye (MB) under UV light. The m-PBI@CNTs/ZnO 1:3 photocatalyst demonstrated the highest degree of degradation of the MB dye (67%) [35].

In this work, for the first time, stable PANI/MWCNT hybrid dispersions were synthesized via in situ oxidative polymerization of aniline in pure formic acid as a dispersion medium and were then used for effective impregnation of microfibrillar cellulose substrates. The relationships between synthesis conditions, structure, and the catalytic properties of methylene blue dye degradation were systematically investigated, with particular emphasis on potential applicability in advanced flexible photocatalytic water purification materials. Unlike conventional PANI/CNT composites, prepared by mechanical blending or diluted aqueous acid dispersion polymerization, the present work introduces a facile strategy for fabricating stable PANI/MWCNT nanodispersions in formic acid, which are suitable for direct fibrous cellulose substrate impregnation. Such an approach is beneficial for the scalable fabrication of flexible conductive and catalytic nanocomposite materials.

## 2. Materials and Methods

### 2.1. Materials

Aniline (≥99%, Merc, Darmstadt, Germany) was distilled under reduced pressure prior to use. Potassium persulfate, K_2_S_2_O_8_ (KPS), hydrogen peroxide (30% H_2_O_2_), formic acid (99%, Merc, Darmstadt, Germany), hydrochloric acid (37% HCl), and absolute ethanol (99%, Fluka, Buchs, Switzerland) were of analytical grade and used as received. Multi-walled carbon nanotubes (MWCNTs; Timestub™ (Chengdu, China) graphitized MWCNTs, TNGM3; purity > 99%, outer diameter: 10–20 nm, length: 5–30 μm, and specific surface area > 80 m^2^ g^−1^) were used as conductive nanofillers. Whatman (Kent, UK) filter paper was employed as the cellulose substrate. Deionized water was used in all experiments.

### 2.2. Synthesis of Carboxylated Polyanilines

Polyaniline was synthesized by oxidative polymerization of aniline in formic acid medium with two kinds of oxidants: KPS and H_2_O_2_. Briefly, 0.152 g of aniline was first dissolved in 14 grams of formic acid in a double-neck round-bottom flask of 20 mL, and then 0.290 g of KPS or 2 mL of 30% H_2_O_2_ was added. Next, the reactant solutions were subjected to a short 2 min ultrasound treatment until full dissolution of the oxidants. In the case of KPS-initiated polymerization of aniline, almost instant blue–green colorization of the reaction media was observed in the first minutes; in the case of H_2_O_2_, an intensive red–violet colorization of the solution was obtained within 1 h. Then, the polymerization reaction was allowed to proceed for six hours at 4 °C in an ice bath. The resulting in situ-carboxilated PANI (c-PANI) emeraldine salt was precipitated in 100 mL of deionized water, glass-filtered, thoroughly washed with deionized water and absolute alcohol to remove any residual oxidant and soluble oligomeric species, and then dried under vacuum at 60 °C overnight. Yield of water-insoluble fraction: 72%.

### 2.3. Preparation of c-PANI/MWCNT Hybrid Dispersions

Two concentrations of hybrid c-PANI/MWCNT dispersions (13 and 26 wt.% content) were prepared via in situ oxidative polymerization of aniline in the presence of MWCNTs. Initially, the required amount of pristine MWCNT powder, 0.02 and 0.04 g resp., was dispersed in two separate glass flasks with 20 mL of formic acid media using short ultrasonication treatment to achieve wetting of MWCNT agglomerates. Then aniline monomer (0.152 g for each batch) was introduced dropwise under continuous stirring, followed by initiation of the polymerization using potassium persulfate (0.290 g) with one minute of ultrasound treatment, which led to almost immediate polymerization. Then, the polymerization reaction was allowed to proceed for one hour at 4 °C in an ice bath under magnetic stirring, resulting in dark-colored fine dispersions of de-agglomerated MWCNTs. The resulting c-PANI/MWCNT hybrids were precipitated and dried following the same procedure as that described for the pure PANI and used for structural characterization or used in pristine dispersion form for direct cellulose impregnation experiments.

When H_2_O_2_ was used as an oxidative initiator, the preparation protocol was similar to that for KPS, except that the polymerization rate was slower. In brief, 2 mL of 30% H_2_O_2_ and 13 wt.% MWCNTs were introduced to the monomer and dissolved in formic acid, and the polymerization was carried out for 12 h at 4 °C in a refrigerator under static conditions, resulting in formation of a very fine dark-violet dispersion.

### 2.4. Impregnation of Cellulose Filter Paper Substrates

Micro-fibrous filter paper cellulose substrates were impregnated with the freshly prepared c-PANI/MWCNT hybrid dispersions using a simple drop impregnation method. The circular filter paper sheets were impregnated dropwise with a plastic syringe pre-filled with c-PANI or c-PANI/MWCNT dispersions under a hood till reaching an even surface soaking, facilitated by the micro-fibrous substrate capillary force intake. The wet impregnated filter paper substrates were then placed on a teflonized hot plate and dried at 90 °C. After drying, the samples were removed and washed thoroughly several times with deionized water and then with absolute alcohol in order to remove any remaining initiator by-products and soluble oligomeric fractions, yielding flexible impregnated cellulose-c-PANI and cellulose-c-PANI/MWCNT samples.

### 2.5. Physicochemical Characterization of Prepared PANI/MWCNT Hybrid Materials

X-ray photoelectron spectroscopy (XPS) studies were performed with a VG ESCALAB II electron spectrometer using AlKα radiation with an energy of 1486.6 eV. The binding energies (BEs) were determined with an accuracy of ±0.1 eV utilizing the C1s line at 285.0 eV (from an adventitious carbon) as a reference. The changes in the chemical composition at the depth of the films were determined on the basis of the areas and binding energies of S2p, C1s, O1s and N1s photoelectron peaks and Scofield’s photoionization cross-sections.

Attenuated total reflection–Fourier-transform infrared (ATR-FTIR) spectra were collected using an IR Affinity-1 spectrophotometer (Shimadzu, Kyoto, Japan) equipped with a MIRacle ATR accessory (diamond crystal, with a depth of penetration of the IR beam into the material of 2 μm).

Raman spectroscopy measurements were carried out using a Thermo Scientific (Waltham, MA, USA) instrument with a 1064 nm laser line. A laser power of 50 mW was used to provide a sufficient signal without increasing the overall fluorescence background or damaging the sample. The optimal exposure time was 5 ms to avoid the thermal degradation of the material. A total of 50 scans were performed.

The surface morphologies of the prepared hybrid materials were studied with scanning electron microscopy (SEM) using a Zeiss Evo 10 microscope (Carl Zeiss Microscopy, Oberkochen, Germany). The photographs were taken in secondary electrons with an accelerating voltage of 25 keV and no conductive coating on the composites. The chemical composition of the surface was investigated with electron-dispersive spectroscopy (EDS) using an Oxford Ultim Max 40 probe (Oxford Instruments, Abingdon, UK). The AZtec software (version 6.1 HF4) was used to compile the results.

Thermal gravimetric analyses (TGAs) were performed using a TGA-4000 Perkin Elmer instrument (Shelton, CT, USA) (temperature range: 40 °C–800 °C; 10 °C/min) in an argon atmosphere.

The hydrodynamic diameter and size distribution of the composite materials were established with a Zetasizer NanoBrook 90Plus PALS instrument (Brookhaven Instruments Corporation, Holtsville, NY, USA) equipped with a 35 mW red diode laser (λ = 640 nm) at a scattering angle of 90°. The sample concentration was 0.5 mg·mL^−1^, and 3 measurements were carried out for each sample at a temperature of 25 °C.

### 2.6. Photocatalytic Study

The photocatalytic activity of the PANI/MWCNT-impregnated cellulose substrates was assessed by using methylene blue dye as a model contaminant. The initial concentration of the dye in an aqueous solution was 5 ppm. The experiments were conducted with a UV-A illumination lamp with maximum emission at 365 nm, 18 W and an illumination intensity of 2.6 mW/cm^2^. The UV irradiation dose for 180 min of exposure was 3.921 × 10^−17^ J/m^2^. The photocatalytic tests were carried out in a semi-batch slurry reactor equipped with two frits blowing tiny bubbles of air in order to saturate the solution in dissolved oxygen using a 3 × 3 cm PANI/MWCNT-impregnated cellulose substrate photocatalyst and 20 mL of the dye solution under a constant stirring rate (400 rpm). The tested systems, the dye solution and the photocatalyst were stirred in the dark for about 40 min before switching on the UV light for 3 h in order to reach an adsorption–desorption equilibrium state. A study of the photocatalytic efficiency of the prepared composites was performed by taking aliquot samples of the dispersion out of the reaction vessel after regular time intervals. The reaction course was monitored by a Cary 4000 UV-Vis Spectrophotometer (Agilent, Santa Clara, CA, USA) in the wavelength range from 200 to 800 nm (λ_max_ = 664 nm for MB). The degree of degradation (DD) of methylene blue was determined using the following equation:

DD = ((C_0_ − C)/C_0_) × 100,

where C_0_ and C are the initial concentration before turning on the illumination and the residual concentration of the dye after illumination, respectively, in the course of the given time interval t.

## 3. Results

### 3.1. Synthesis of Carboxylated PANI/MWCNT Dispersions in Formic Acid

**Scheme 1:**
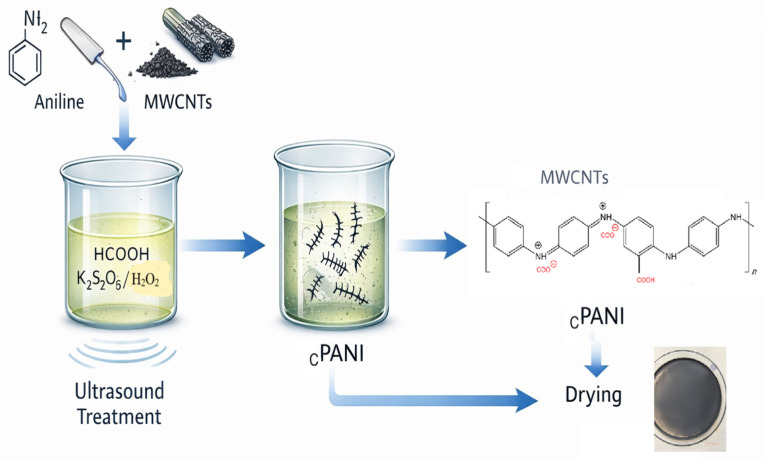
Preparation of in situ c-PANI surface-stabilized MWCNT dispersions in FA.

The aim of this study was to validate the in situ aniline oxidative polymerization approach in pure formic acid (FA) in the presence of pristine multi-walled carbon nanotubes in order to prepare stable carboxylated polyaniline surface-wrapped MWCNT dispersions suitable for impregnation of fibrous substrates, such as cellulosic filter paper, as well as to investigate their photocatalytic degradation properties using methylene blue as a model dye pollutant. Formic acid was employed as both the polymerization medium and dispersion stabilizer, thus facilitating the MWCNTs’ debundling and strong interfacial interactions between the MWCNTs and the growing PANI chains, as illustrated in Scheme 1.

Other beneficial virtues of the use of formic acid as a polar organic molecule dispersant and polymerization medium are the relatively high volatility and good solubilization ability for PANI. These properties of FA allow efficient drying and smooth impregnation of various high-surface substrates, e.g., fibrillary-glass- or cellulose-based substrates. Another interesting feature resulting from the polymerization of aniline in FA is the partial carboxylation and sulfonation of the PANI main chain, which was described in the fundamental work by Trchová et al. [5]. The as-obtained carboxylated PANI emeraldine salt can be considered an amphiphilic macromolecule, which gives wide possibilities for both inter- and intramolecular polymer–polymer and polymer–inorganic filler interface interactions based on acid–base, aromatic π–π and H-bonding. Pristine PANI in both emeraldine base and protonated acid-doped form has already shown a good ability to surface wrap pristine multi-walled carbon nanotubes, which is particularly pronounced when using oxidized (carboxylated) MWCNTs, where specific ionic interactions between polymers and CNTs at the interface take place [18,36,37]. In most of the published works on the solution-based in situ hybridization of PANI@CNTs, the oxidative polymerization of aniline was performed using a classical aqueous hydrochloric acid solution, where the intrinsic bad dispersibility of the hydrophobic CNTs is partially improved by either their surface oxidation or by adding another hydrophilic polymer for the CNTs’ surface modification. However, the polymerization of aniline in aqueous media usually leads to the formation of intrinsically hydrophobic PANI@CNT hybrid particles, which quickly agglomerate and settle down, making their use as individual particles difficult. In other words, this limitation hampers the preparation of colloidally stable PANI-wrapped CNT dispersions for bulk or surface impregnation and coating of different substrates and for imparting beneficial properties, such as increased electrical conductivity, the EMI shielding effect, and sensor and catalyst activity. Therefore, the use of concentrated formic acid for the polymerization of aniline in the presence of pristine MWCNTs is especially beneficial, as the in situ-formed carboxylated-PANI@CNT hybrid particle dispersions showed very good colloidal stability even at a relatively high MWCNT concentration of 2 mg·mL^−1^.

**Figure 1:**
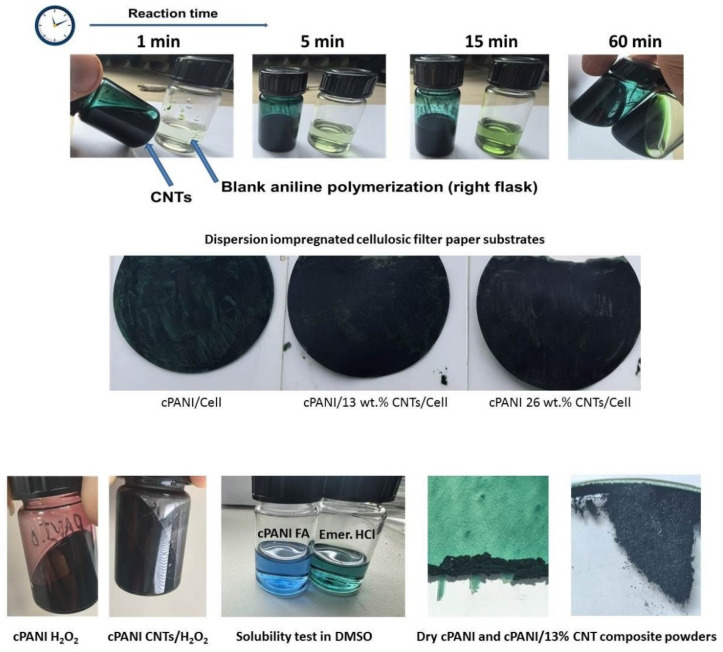
Illustration of the c-PANI @ c-PANI/MWCNT polymerization process with KPS or H_2_O_2_ as oxidizing agents (**upper row**); the c-PANI dispersion-impregnated cellulose substrates (**middle**) as well as pictures of the solubility test of the obtained c-PANI in DMSO and the dry, pure c-PANI and c-PANI/13% MWCNT composite powders (**down**).

In this work, hybrid c-PANI dispersions containing 13 and 26 wt.% MWCNTs were obtained by adjusting the pristine MWCNTs’ loading into the in situ-formed aniline formate salt. Potassium persulfate was primarily used as an initiator due to its ability to promote the formation of the electrically conductive emeraldine formate salt form of high-molecular-weight c-PANI. Hydrogen peroxide was also examined as an oxidative agent; however, it caused the formation of a high fraction of low-molecular-weight 2,5-diamino-p-benzoquinone-like oligomeric species, consistent with previous reports [38,39]. The polymerization of aniline, with and without MWCNTs, was facilitated by short ultrasound treatment/homogenization at room temperature, which was stopped once the solutions began to darken, indicating oxidative polymerization reactions. Ultrasound treatment positively affected the polymerization kinetics, the dissolution/disproportionation of KPS and the dispersibility of the initially agglomerated MWCNTs. It was observed that in the case of KPS polymerization, the presence of MWCNTs significantly shortened the initiation time of the reaction (only one minute) and the ongoing propagation step, compared with the much slower bulk aniline polymerization, as shown in Figure 1.

The increase in the MWCNT content from 13 to 26 wt.% reduced the initiation time, which was an indication of an anisotropic-substrate-driven effect. This fact could be partially explained by the local overheating of the CNTs’ nanophase and its ability to instantaneously anchor the propagating c-PANI chains based on the above-mentioned surface interactions. Another interesting observation was the color of the final pristine c-PANI_KPS_ (emeraldine PANI form: green) and c-PANI_KPS_/MWCNT dispersion (fully oxidized pernigraniline PANI form: blue–green). In the case of c-PANI_H2O2_, the color of the reaction solution was vinegar red, which was also an indication of the formation of high-oxidation pernigraniline PANI [40]. After drying and purification, the isolated c-PANI_KPS_ powder was readily dissolved in DMSO solvent, forming a blue-colored transparent solution (2 mg·mL^−1^). The sample did not discolor after filtering through a 0.1 µm PTFE syringe filter, which is typical for a polymer solution. Unlike c-PANI_KPS_, the commercial PANI emeraldine HCl salt had limited dissolution in DMSO and green colorization. Moreover, the solution notably paled off after nanofiltration, which was an indication of fractionation due to chain agglomeration (the solution did not contain only individual macromolecules). Interestingly, the as-obtained polymerization solutions of c-PANI in FA significantly discolored after filtering, which can be attributed to nanoparticle formation. This fact revealed that FA has a reduced ability to dissolve c-PANI.

In the next step, the as-obtained solutions/dispersions of both c-PANI and c-PANI/MWCNTs were used for impregnation and coating of microfibrillar cellulosic filter paper substrates by dip coating. The high volatility of the FA dispersant media was exploited to achieve an even impregnation of the cellulose substrate followed by quick drying. Finally, the impregnated cellulose samples were subsequently washed with absolute alcohol and immersed in distilled water overnight to remove the impurities formed as by-products during the polymerization (sulfuric acid and potassium sulfate salt; oligomers).

**Figure 2:**
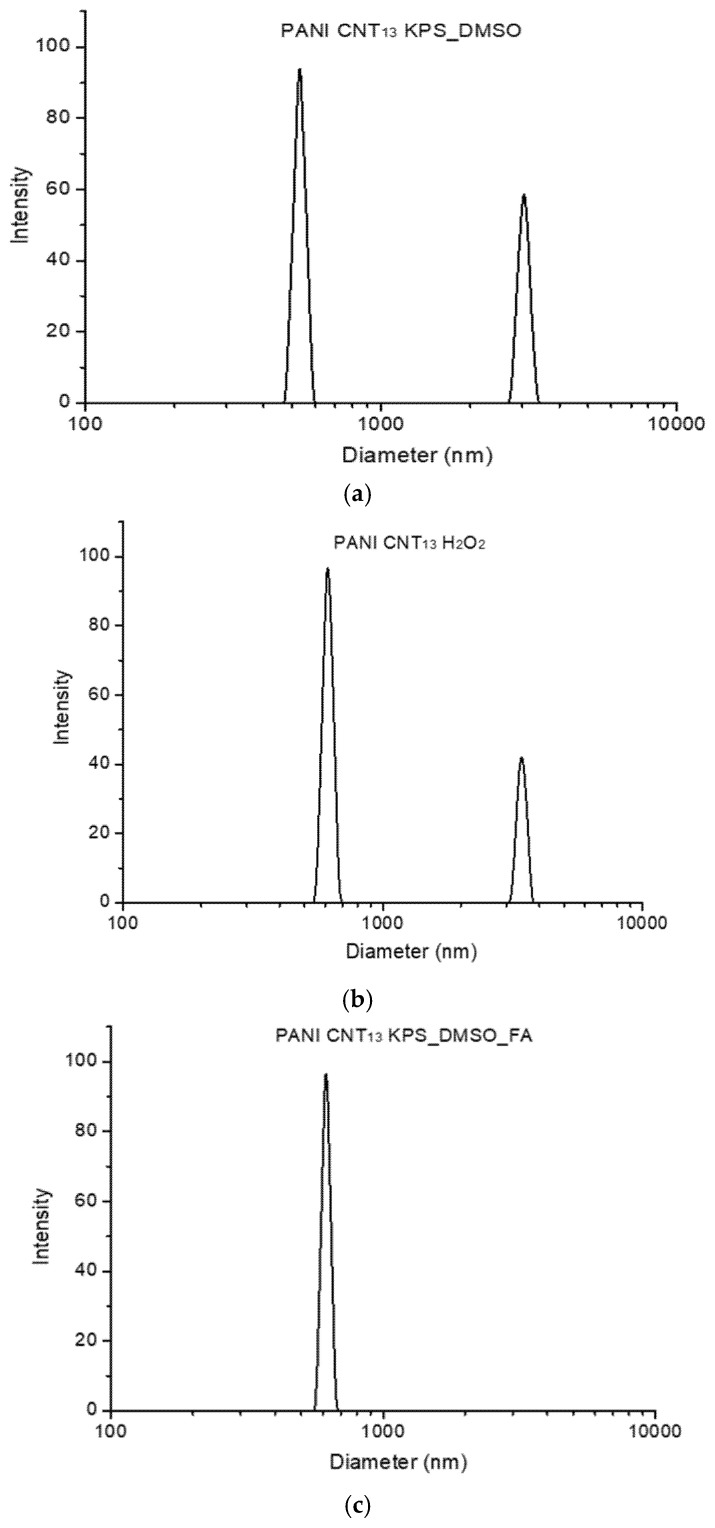
DLS measurement of redispersed c-PANI_KPS_/CNTs 13 wt.% (**a**) and c-PANI_H2O2_/CNTs 13 wt.% (**b**) in DMSO and c-PANI_H2O2_/CNTs 13 wt.% in DMSO/FA solvent (**c**). The total concentration of c-PANI/CNTs was 0.5 mg·mL^−1^.

The colloidal stability of the c-PANI-wrapped MWCNT dispersions was further evaluated by dynamic light-scattering (DLS) analysis. The isolated c-PANI_KPS_/MWCNTs and c-PANI_H2O2_/MWCNTs in the form of ammonia-neutralized dried powders were redispersed in DMSO and mixed DMSO/FA (9:1) solvents using mild ultrasonic treatment and analyzed by DLS (Figure 2).

**Figure 3:**
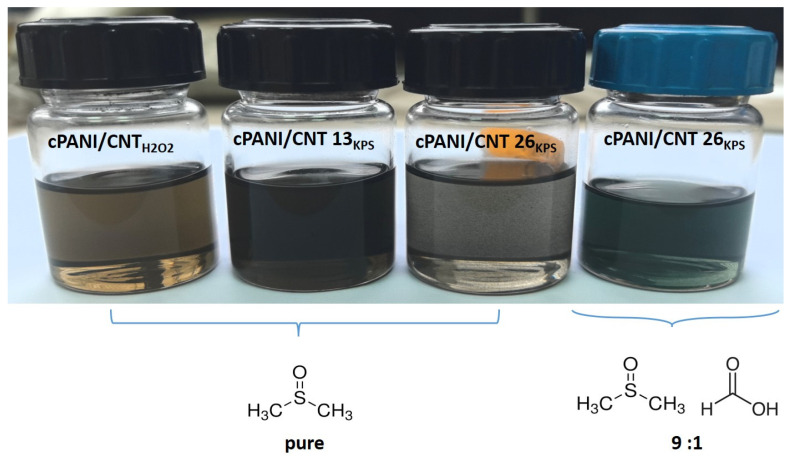
Digital images of redispersed c-PANI/CNT formulations in pure DMSO and mixed DMSO/FA solvents.

The DLS measurement demonstrated a relatively good colloidal stability of the systems in the polar organic media used. Each dispersion was measured three times within 4 min, and the results (particle size distribution plots) were identical. A dominant peak, at about 660–670 nm, was observed for the three samples of redispersed c-PANI/CNT composite powders, which was probably due to the scattering of individual hybrid nanoparticles. In the case of the c-PANI_KPS_/CNTs 13 and c-PANI_H2O2_/CNTs 13 dispersions in DMSO, a second, less intense peak at about 3 microns was detected. Obviously, the system tended to form some larger agglomerates in DMSO, but their fraction was negligible. When DMSO/FA was substituted for pure DMSO, the second peak was not detected, as illustrated for the c-PANI_H2O2_/CNTs 13 dispersion (Figure 2c). This observation clearly showed the stabilization role of the FA solvent, used as a reaction medium, and the corresponding emeraldine salt for the good colloidal stability of the pristine reaction dispersions of hybrid c-PANI/MWCNT particles. The dried sample with a high MWCNT content (c-PANI_KPS_/MWCNT 26) was also redispersed in DMSO; however, a notable fraction of agglomerated particles was observed by the naked eye, as illustrated from digital images of the different dilute hybrid dispersions shown in Figure 3. The observed color change of the c-PANI_KPS_/MWCNT hybrid dispersions in pure DMSO and in a mixed 9:1 DMSO/FA solvent from dark brown to green was attributed to the structural transformation of the emeraldine base into a protonated emeraldine salt of c-PANI. Notably, the use of an FA-containing co-solvent mixture enabled fine redispersion of the sample with a high CNT content (c-PANI_KPS_/MWCNT 26), which, as mentioned before, showed poor dispersibility in pure DMSO. This property could be attributed to the lower c-PANI-to-CNT wt. ratio, which affected the surface wrapping efficiency by shielding the π–π stacking of bare CNT surfaces from one side and the specific intramolecular hydrophobic and ionic interactions of c-PANI and polymer–solvent interactions of the emeraldine base and the emeraldine FA salt, respectively.

**Figure 4:**
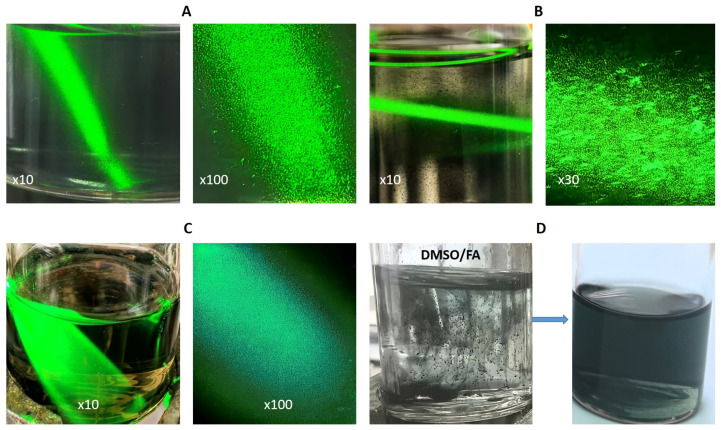
Digital images of green-laser light-scattering effect of c-PANI_KPS_/CNT 13 (**A**), c-PANI_KPS_/CNT 26 (**B**), and c-PANI_H2O2_/CNT 13 (**C**) hybrids in DMSO; illustration of spontaneous redispersion of c-PANI_KPS_/CNT 26 powder in contact with mixed DMSO/FA solvent (**D**).

The stability of the c-PANI/CNT dispersions was further evaluated by a green-laser (λ = 532 nm) light-scattering visualization experiment, as shown in Figure 4. The dispersions of all redispersed composite powders showed the typical coherent light particle scattering Tindall effect, with discrete observation of chaotic Brownian motion of individual particles at a higher camera magnification (see also the video file in the Supplementary Materials). In the case of the c-PANI_KPS_/MWCNT 26 dispersion in DMSO, both micronized, agglomerated and smaller anisotropic structures were visible at ×30 magnification, while only individual particles were observed for c-PANI_KPS_/CNT 13 at ×100 magnification (Figure 4A,B).

The finest particle dispersion was observed in the case of the c-PANI_H2O2_/CNTs 13 dispersion, with an interesting veil-like light-scattering effect when the laser beam was directed perpendicular to the dispersion boundary surface (Figure 4C). The process of spontaneous redispersion of the hybrid material, containing 26 wt.% MWCNTs (c-PANI_KPS_/MWCNT 26 powder), immersed in mixed 9:1 DMSO/FA solvent, is also documented in the digital images presented in Figure 4D.

### 3.2. Characterization of c-PANI/MWCNT-Impregnated Cellulose Substrates

#### 3.2.1. SEM and EDS Mapping/Spectral Analysis

**Figure 5:**
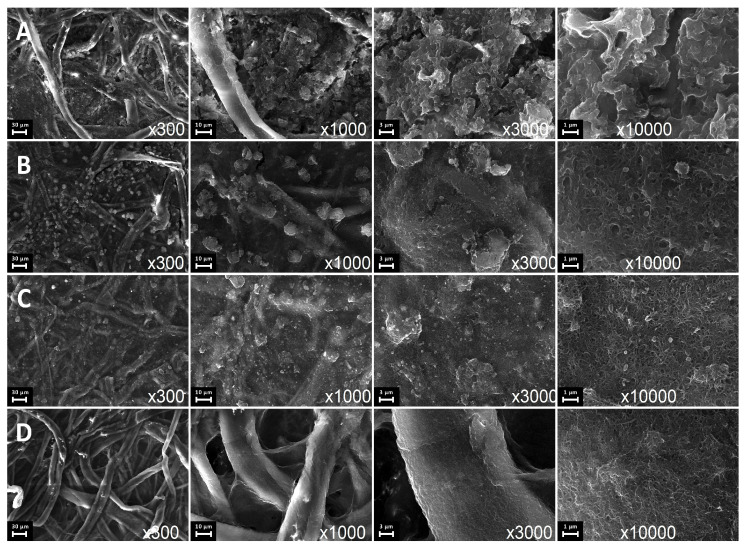
SEM images of c-PANI_KPS_-0 (**A**); c-PAN_KPS_/CNT-13 (**B**); c-PANI_KPS_/CNT-26 (**C**); and c-PANI_H2O2_/CNT-13 hybrids deposited on cellulose filter paper (**D**).

The morphology, composite structure and degree of c-PANI/MWCNT deposition onto microfibrous cellulose substrates were studied by SEM analysis. Figure 5 displays the SEM images of the used filter paper substrate surfaces impregnated with the freshly synthesized c-PANI and the different c-PANI/MWCNT dispersions in FA. The SEM analysis of pristine c-PANI_KPS_-0 (Figure 5A) showed typical sponge-like deposition of coalescent PANI dry mass, with some clustering morphology. Some individual cellulose microfibrils with a thin PANI coating were also seen, which is rarely observed in typical water-based PANI formulations. The SEM of c-PAN_KPS_/CNT-13- and c-PAN_KPS_/CNT-26-impregnated cellulose (Figure 5B,C) showed an interesting mixed population of both individual micron-sized c-PANI particles, with a sponge-like morphology, and a PANI-matrix-incorporated CNT network evenly covering the cellulose microfibrils. The presence of deposited pristine c-PANI microparticles could be explained by some bulk (CNT substrate-independent) polymerization of aniline in the reaction mixture. This suggestion is supported by the fact that a decrease in particle size and density was found with an increasing CNT content from 13 to 26 wt.% (with respect to the monomer) in the reaction mixture. Another proof of this hypothesis is the increased bareness of the deposited MWCNT network with the twofold increase in the nanotube content, from bulk c-PANI-matrix-embedded CNTs to a more CNT-exposed structure.

**Figure 6:**
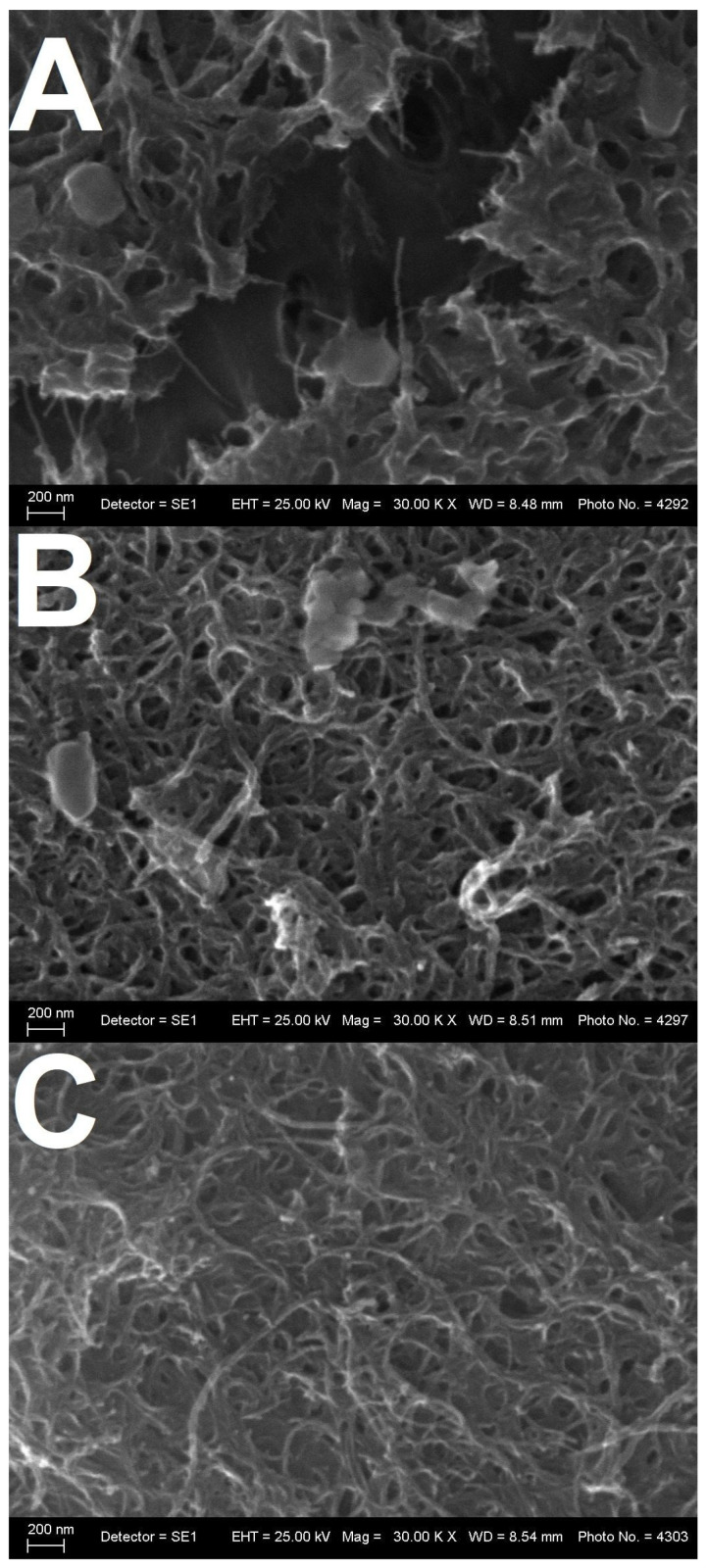
SEM images of c-PAN_KPS_/CNT-13 (**A**); c-PANI_KPS_/CNT-26 (**B**); and c-PANI_H2O2_/CNT-13 hybrid composites (**C**).

In the case of the c-PANI_H2O2_/CNT-13 hybrids, there was the most even CNT network deposition onto the individual cellulosic microfibrils with the barest CNT structure (less excessive PANI deposition) without deposition of any PANI-based particles. This observation could be explained by the specific H_2_O_2_-induced aniline polymerization in FA with predominant oligomeric fractions (soluble in ethanol and water), which was washed out during the solvent purification procedures, whereas the deposited dried CNTs remained fixed onto the cellulose substrate. The high-magnification SEM images of the three cellulose-deposited c-PANI/CNT composites shown in Figure 6 reveal, in much detail, the above-discussed observations for the different kinds of CNT/c-PANI hybridization, depending on the reaction conditions.

There was an obvious transition from a seemingly bulk-like composite for the cPAN_KPS_/CNT-13 coating, where individual and intact CNTs were embedded in the PANI matrix (Figure 6A), to barer CNTs@PANI wrapped network deposition for the c-PANI_KPS_/CNT-26 and c-PANI_H2O2_/CNT-13 hybrids (Figure 6B,C).

**Figure 7:**
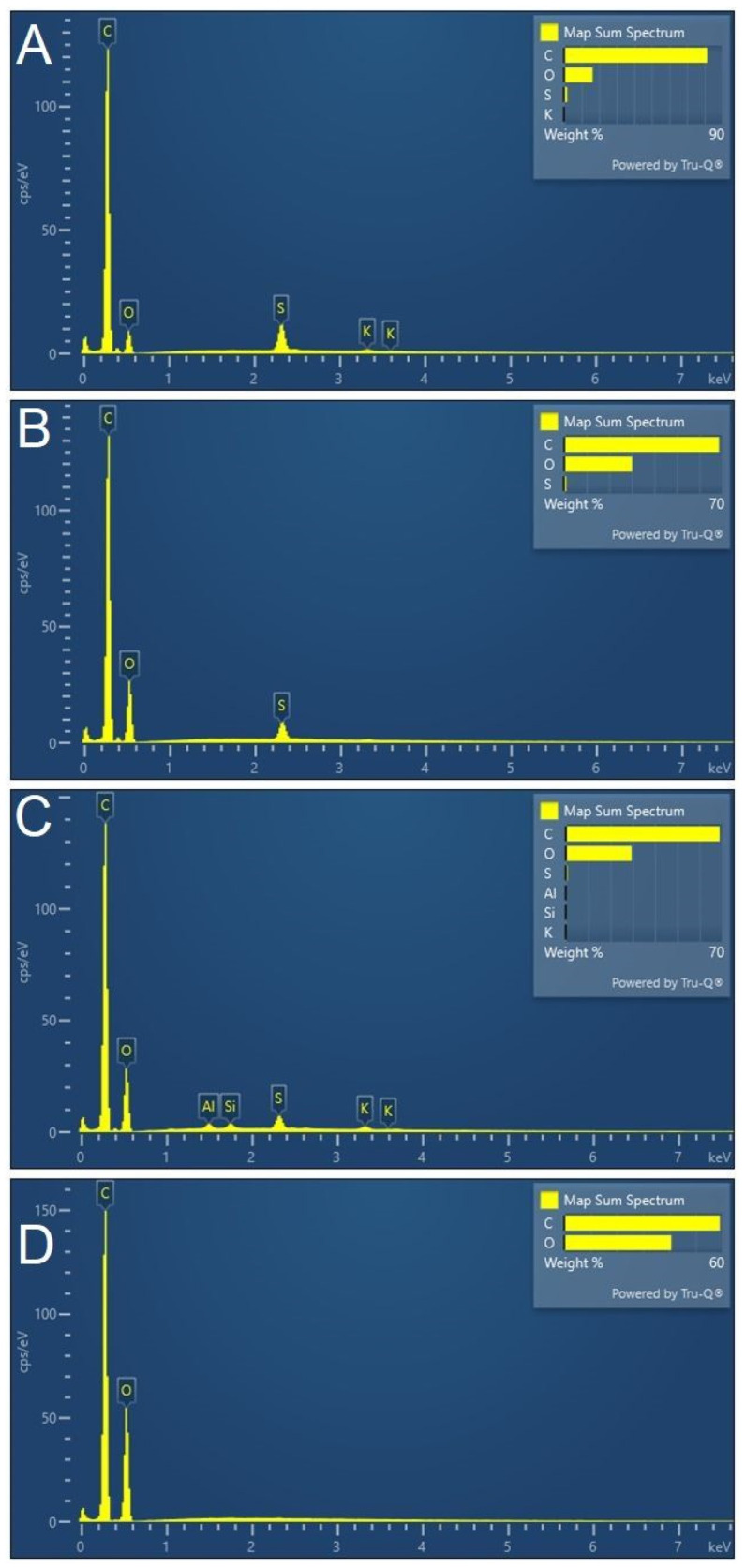
EDS spectra of c-PANI_KPS_-0 (**A**); c-PAN_KPS_/CNT-13 (**B**); c-PANI_KPS_/CNT-26 (**C**); and c-PANI_H2O2_/CNT-13 composite powders (**D**).

The elemental compositions of the isolated and purified c-PANI_KPS_-0, c-PANKPS/CNT-13, c-PANI_KPS_/CNT-26 and c-PANI_H2O2_/CNT-13 hybrids in the form of powders were revealed by their EDS spectra, as shown in Figure 7.

In the three samples of cellulose-impregnated cPANI formulations, synthesized by KPS-mediated polymerization of aniline in FA, there were minor signals for detected sulfur and, except for the c-PANI_KPS_/CNT-13 sample, some traces of potassium. In the c-PANI_H2O2_/CNT-13 composite, only C and O were detected. The presence of elemental sulfur could be attributed to PANI ion-exchange-bonded sulfuric acid as a by-product of the KPS disproportion reaction in FA and/or covalently bonded by the sulfonation side reaction. The observed decrease in the elemental C content and the increase in the O content in the CNT-containing hybrids (Table 1) could be attributed to both formate-doped PANI, with possible partial carboxylation, and the possible surface oxidative functionalization of CNTs with –OH and –COOH groups, which requires further detailed analysis and investigation of isolated de-hybridized CNTs by filtration or centrifugation from strong polar but unoxidative solvents, like methansulfonic or trifluoroacetic acid, in order to remove the surface-wrapped c-PANI. The presence of Al and Si in c-PANI-_KPS_/CNT-26 could be attributed to contamination.

#### 3.2.2. Thermal Gravimetric Analysis

**Figure 8:**
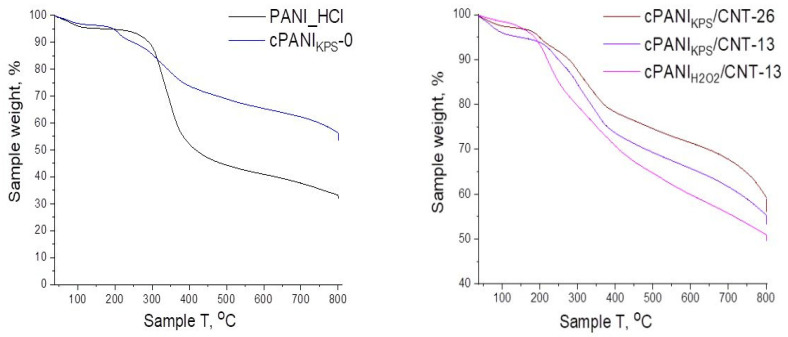
TG curves of PANI_HCl; c-PANI_KPS_-0; c-PANI_H2O2_/CNT-13; c-PANI_KPS_/CNT-13; and c-PANI_KPS_/CNT-26.

The thermal stability of the synthesized materials was evaluated by thermal gravimetric analysis (TGA), as presented in Figure 8. The TGA curve of commercial PANI_HCl shows a multistep degradation process. The initial weight loss below 150 °C was attributed to the removal of physically adsorbed moisture [35]. A minor mass loss between 150 and 300 °C was associated with the release of loosely bound dopant species. The most significant degradation occurred in the range of 300–400 °C, corresponding to the decomposition of the polymer backbone [41,42]. Above 400 °C, a gradual weight loss was observed, related to carbonization and further structural breakdown, resulting in a residual mass at approximately 800 °C.

The TGA plot of c-PANI_KPS_-0 shows improved thermal stability compared with PANI_HCl, with a more gradual weight loss profile and a significantly higher residual mass. The absence of a sharp degradation step suggested a more stable polymer structure, likely due to the differences in the polymerization mechanisms and molecular organization due to intramolecular polymer–electrolyte complex (acid–base) formation from c-PANI macromolecule chains. The reduced mass loss at lower temperatures also indicated lower moisture and volatile compound contents [3]. Significant carbonization residue was observed for both the PANI_HCl and c-PANI samples above 400 °C (35 and 55% resp.), which could be attributed to the inert atmosphere (argon was used instead of oxygen). It must be noted that in an oxygen atmosphere, almost full carbon residue burning (from both pristine PANI and for the c-PANI/CNT composites) occurs at 800 °C [43].

The incorporation of carbon nanotubes further enhanced the thermal stability of the materials. All c-PANI/CNT composites demonstrated reduced total mass loss and higher residual mass compared with the pristine polymer [44].

The sample prepared with H_2_O_2_ (c-PANI_H2O2/_CNT-13) exhibited the highest total mass loss, which was in accordance with the finding that, in this sample, mainly an individually wrapped cPANI/CNT hybrid structure existed, isolating the polymer bulk composite structure of the cPANI_KPS_ hybrid powders with the higher remaining c-PANI matrix.

Overall, the TGA results confirm that both the polymerization approach and the incorporation of MWCNTs play a crucial role in the thermal behavior of PANI-based materials, with the c-PANI_KPS_/CNT composites showing the most promising thermal stability.

#### 3.2.3. FT-IR Investigations

**Figure 9:**
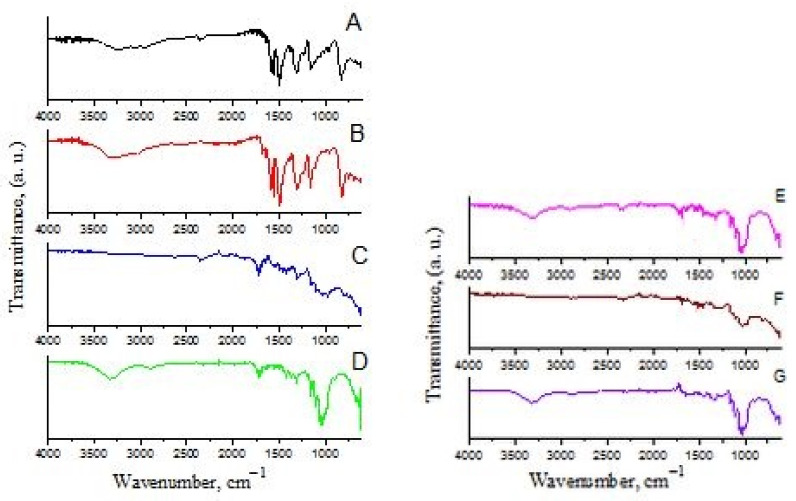
FT-IR spectra showing the characteristic vibrational bands of polyaniline and the effect of the oxidant type and CNT content on the chemical structure of the nanocomposites: (**A**)—PANI_HCl; (**B**)—cPANI_KPS_-0 base; (**C**)—cPANI_KPS_-0; (**D**)—cPANI_H2O2_-0; (**E**)—cPANI_H2O2_/CNT-13; (**F**)—cPANI_KPS_/CNT-13; and (**G**)—cPANI_KPS_/CNT-26.

FT-IR spectroscopy was employed to investigate the chemical structure and interfacial interactions in the c-PANI@MWCNT-impregnated cellulose substrates. As shown in Figure 9, all samples exhibit the characteristic absorption bands of polyaniline, confirming the formation of the emeraldine salt structure. The broad band observed at 3200–3400 cm^−1^ is attributed to the stretching vibrations of N–H groups in amine and imine units, partially overlapping with O–H stretching vibrations of cellulose and oxygen-containing functional groups [3,5,45]. The bands located at ~1580–1600 cm^−1^ and ~1480–1500 cm^−1^ correspond to the C=C stretching vibrations of quinoid and benzenoid rings, respectively, which are typical features of conductive PANI [3,5,46]. The peak at ~1290–1310 cm^−1^ is assigned to C–N stretching of secondary aromatic amines, while the characteristic band at ~1140 cm^−1^ (polaron band) indicates the protonated and electrically conductive state of PANI [3,5]. Compared with cPAN_IH2O2_-0 (Figure 9A), cPANI_KPS_-0 (Figure 9B) exhibits more pronounced quinoid and polaron bands, suggesting a higher oxidation and protonation degree induced by the stronger oxidizing agent, KPS [5,46]. After incorporation of MWCNTs (Figure 9C–E), noticeable band broadening and slight peak shifts are observed, particularly in the N–H/O–H stretching region, indicating strong interfacial interactions between PANI chains and the MWCNT surface. In addition, a band near ~1590 cm^−1^ can be assigned to the IR-active phonon mode of nanotubes. The appearance of bands at approximately ~1720 cm^−1^ is attributed to the stretching vibrations of carboxylic acid groups (–COOH), corresponding to C=O and C–O stretching modes [46,47,48,49,50,51]. To exclude the overlapping signal from ion-bonded formate anions, the samples were subsequently treated with concentrated ammonia and then washed with water and re-acidified with a 1 M HCl solution, by which the ammonium formate salt was washed out, and only the covalently bonded –COOH groups on the aromatic ring structure remained. The absorbance for S-O symmetric str. vibration at 1080 cm^−1^ and S-O asym. vibration at ~1190 cm^−1^ observed in the cPANI samples obtained with KPS as the oxidizing agent could be attributed to the presence of covalently sulfonated adducts as a side reaction but could also be the result of the remaining cPANI ion-bonded sulfuric acid, which was not displaced by the ammonia treatment and needed a stronger base, e.g., NaOH.

With an increasing CNT content, the intensity of the bands related to oxygen-containing groups slightly increased, while some characteristic PANI bands became less intense due to the higher contribution of CNTs and stronger interfacial coverage [39,47]. Compared with pristine PANI, small peak shifts and band broadening further supported the existence of strong π–π interactions between the conjugated PANI chains and the graphitic structure of CNTs [8,48]. Moreover, changes in the O–H stretching region of cellulose indicated hydrogen bonding between the cellulose, PANI, and functionalized CNTs, confirming successful impregnation and good interfacial compatibility within the hybrid substrate [8]. These structural features are favorable for charge transfer processes and are considered responsible for the improved photocatalytic dye removal performance of the PANI/CNT nanocomposites [52,53].

#### 3.2.4. Raman Study

**Figure 10:**
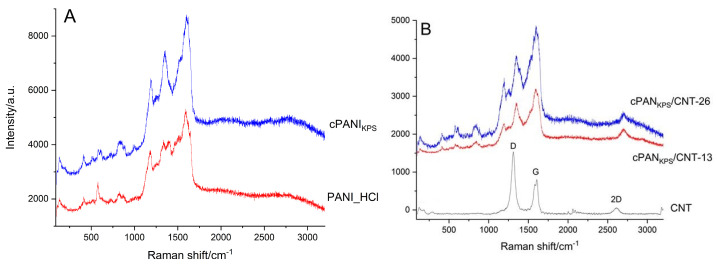
Raman spectra of (**A**) commercial PANI_HCl and c-PANI_kps_ and (**B**) c-PANI@MWCNT-impregnated cellulose substrates (c-PANI_KPS_).

Figure 10A,B present the Raman spectra of the synthesized c-PANI; commercial PANI_HCl; MWCNTS; and prepared c-PANI@MWCNT-impregnated cellulose substrates.

The Raman spectra of the commercial PANI_HCl and cPANI_KPS_ (Figure 10A) are similar and exhibit several characteristic vibrational bands associated with the benzenoid and quinoid structures of the polymer [54,55]. A weak band observed at around 576 cm^−1^ is attributed to benzene ring deformation, while the peak near 869 cm^−1^ corresponds to C-H out-of-plane vibrations of the aromatic ring. The very strong bands at ~1185 cm^−1^ and ~1330–1395 cm^−1^ are assigned to C-H in-plane bending coupled with C-N stretching and C-N^+^ stretching vibrations associated with protonated imine units (polaron structure), respectively. The prominent band at 1592 cm^−1^ corresponds to C=C stretching in the benzenoid rings.

The Raman spectrum of the carbon nanotubes (Figure 10B) reveals a disorder-induced band (D-band), centered at 1312 cm^−1^, which splits into two peaks at 1309 and 1318 cm^−1^. The spectrum also exhibits peaks at 1580, 1586, and 1606 cm^−1^. The peaks at 1580 and 1586 cm^−1^ correspond to the G-band (in-plane sp^2^ graphitic vibration), while the peak at 1606 cm^−1^ is attributed to the defect-activated D-band. The 2D (or G)-band, corresponding to a second-order overtone of the D-band, is observed at around 2611 cm^−1^. The splitting and broadening of the bands, together with a high D/G intensity ratio (>1), indicate a significant degree of structural disorder and defects in the MWCNTs. The presence of both D and D′-bands further indicates a high defect density [56,57,58]. The Raman spectra of the cPANI/MWCNTs composites (Figure 10B) are dominated by PANI vibrational bands, while the characteristic CNT bands (D, G and 2D) appear with comparatively lower intensities. The 2D band exhibits a shift to higher wavenumbers (~2700 cm^−1^), which may suggest strong π–π interactions and possible interfacial charge transfer between the polymer matrix and carbon nanotubes [18,59,60,61]. The upshifts of the D- and G-bands further support electronic interaction between the PANI and MWCNTs in the composite [62].

#### 3.2.5. XPS Investigations

**Figure 11:**
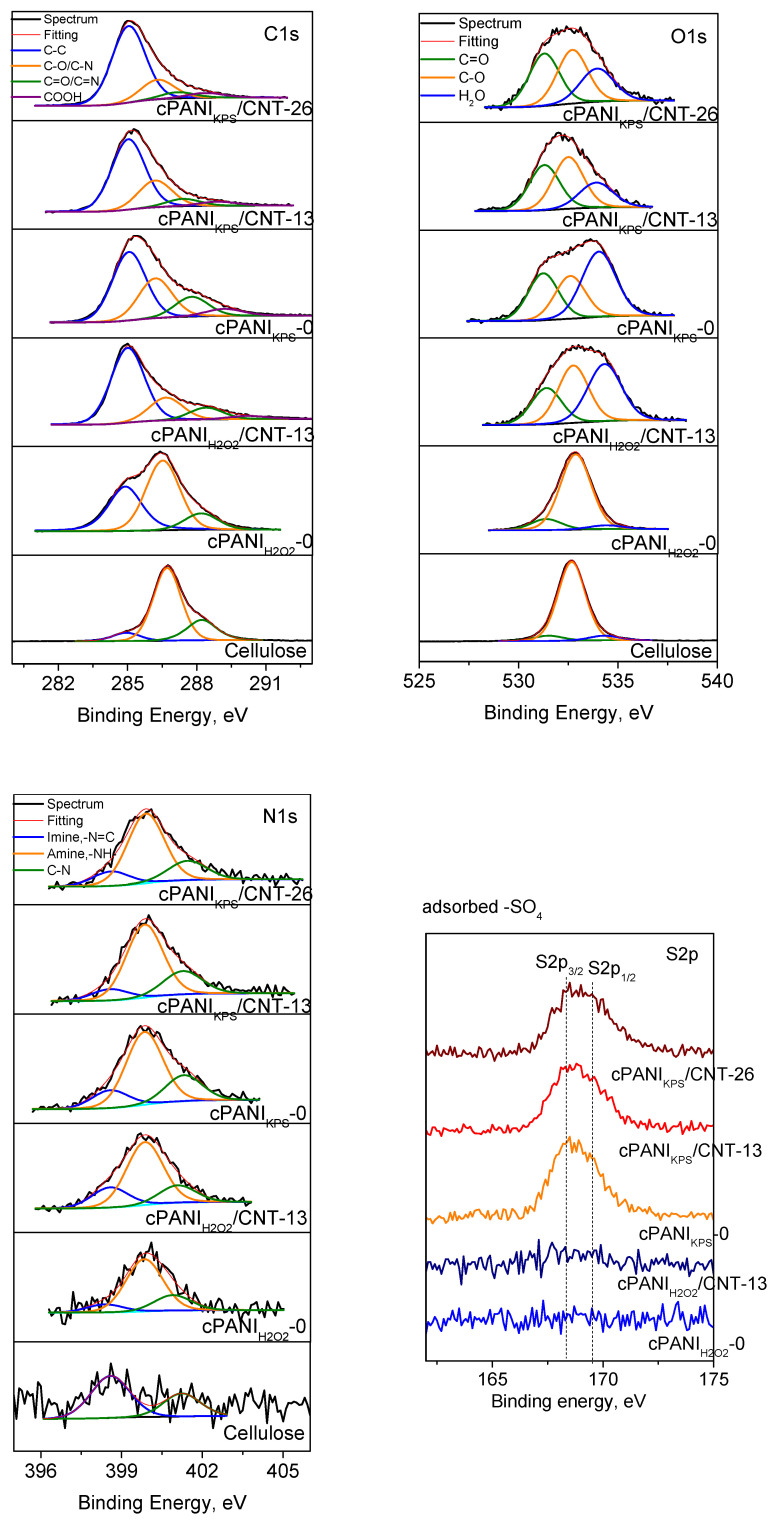
Deconvoluted photoelectron spectra of C1s, O1s, and N1s and core-level spectra of S2p of c-PANI_H2O2_-0; c-PANI_KPS_-0; c-PANI_H2O2_/CNT-13; c-PANI_KPS_/CNT-13; and c-PANI_KPS_/CNT-26 hybrid composites.

The XPS results show that the chemical elements C, O, N and S were registered on the surface. The differences in the surface compositions (Table 2) and electronic structures of the powders obtained with polymers were investigated. The interpretation of the obtained C, O and N spectra shows that their shape is asymmetric, which led to their decomposition with Lorentzian–Gaussian curve fitting (Figure 11). The deconvoluted C1s spectra show several peaks assigned to C-C, C-O/C-N, C=O/C=N and COOH bonds (Table 3) [63].

A difference in the shape of the spectra is observed for the samples prepared with hydrogen peroxide. The PANI-13 sample shows a larger area of the peak corresponding to the C-C bond and a smaller area of the peak associated with the C-O/C-N bonds compared with the PANI-0 sample. Samples treated with potassium persulfate show similar spectra, with an observed trend of decreasing peak areas associated with C-O/C-N, C=O/C=N and COOH bonds. Pure cellulose shows a low-intensity peak of random carbon, a peak attributed to carbonyl groups, and a high-intensity peak corresponding to the bond of carbon to O and N. The spectra of O1s are broad and confirm the bonds observed for C1s [64]. In PANI-0 and pure cellulose, the C-O peak stands out, while in the other samples, this peak has the smallest area. The spectrum of nitrogen shows imine, amine, and C-N bonds. The sulfur is in the form of sulfates adsorbed on the surface. The presence of sp^2^ carbon (284.4 ÷ 284.7 eV) corresponding to the CNTs is not registered because of PANI wrapping the CNTs, as confirmed by SEM analysis.

### 3.3. Photocatalytic Study of Prepared Carboxylated PANI/MWCNT-Impregnated Cellulose Substrates

Industrial dyes are among the major chemicals that render water unsuitable for consumption. Methylene blue is known for its carcinogenic, toxic, and non-biodegradable nature and can cause a severe threat to human health and environmental safety. It is often discharged into natural water bodies, creating hazards for humans and other living organisms. MB is a well-known cationic and primary thiazine dye with the molecular formula C1_6_H_18_N_3_ClS. It is an aromatic heterocyclic basic dye [65]. For these reasons, this dye was chosen as a model pollutant in our studies.

**Figure 12:**
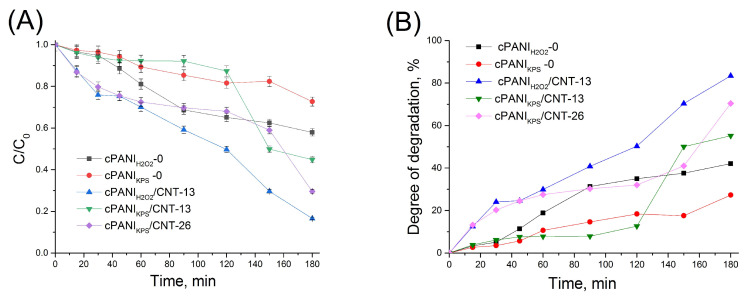
(**A**) The concentration changes in methylene blue dye vs. time of UV light and (**B**) degree of degradation with c-PANI_H2O2_/CNT-13; c-PANI_KPS_/CNT-13; c-PANI_KPS_/CNT-26; c-PANI_H2O2_-0; and c-PANI_KPS_-0 photocatalysts.

**Figure 13:**
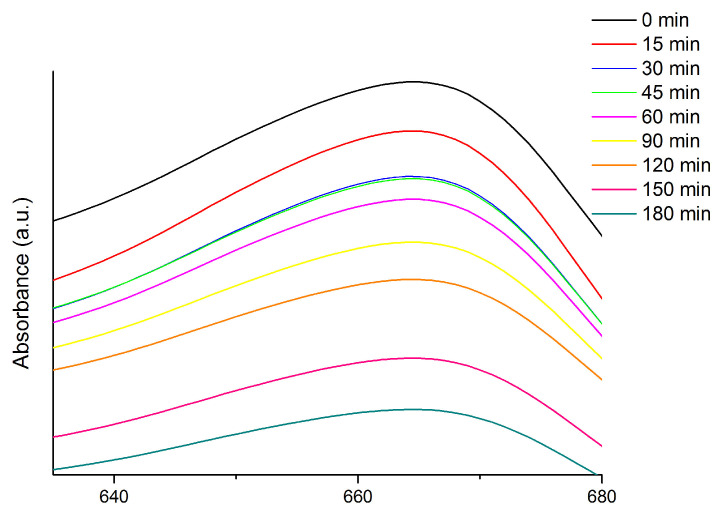
UV–vis absorption spectra of MB dye during irradiation time period using c-PANI_H2O2_/CNT-13 as the photocatalyst.

The photocatalytic ability of the synthesized carboxylated PANI/MWCNT hybrid composites was investigated in the photocatalytic degradation of methylene blue dye as a model contaminant in aqueous solutions. Figure 12A,B show the concentration changes of methylene blue dye during the photocatalytic degradation reaction under UV illumination as well as the degree of degradation. Figure 13 presents the UV–vis absorption spectra of methylene blue dye during the irradiation time period using c-PANI_H2O2_/CNT-13 as the photocatalyst. The highest degree of degradation of the methylene blue dye was achieved in the presence of the c-PANI_H2O2_/CNT-13 hybrid photocatalyst (83%). This result could be explained by the evenly deposited bare surface of the CNT network, as observed by SEM analysis. The Raman study of this composite sample confirmed the significant structural disorder and many defects in the MWCNTs, which had a positive effect and improved the photocatalytic activity of the carboxylated PANI/MWCNTs [37]. The composites c-PANI_KPS_/CNT-26 and c-PANI_KPS_/CNT-13 demonstrated lower degradation values: 70% and 55%, respectively. Increasing the content of CNTs up to 26 wt.% during preparation of the composite led to enhanced photocatalytic efficiency (70% degradation). A similar result was observed by Qurtulen Qurtulen et al. [31] in the degradation of Brilliant Green dye using PANI/CQD nanocomposite photocatalysts with different contents of carbon quantum dots.

The photocatalytic results show that the carboxylated PANI/MWCNT-impregnated cellulose substrates possessed improved photocatalytic activity (55–83%) compared with that of pure PANI (27–42%). The rate constants of the c-PANI_H2O2_/CNT-13 and c-PANI_KPS_/CNT-26 increased above 50%, compared with that of the pure c-PANI_H2O2_-0 and cPANI_KPS_-0 (Table 4). According to other research groups, the photocatalytic ability of pure PANI could be due to the π–π* transition, wherein the excited-state electrons were transferred from the π to the π* orbital [34,66]. The enhanced photocatalytic activity of the synthesized PANI/MWCNT hybrid materials could be assigned to the synergistic effect between PANI and carbon nanotubes, which improved the light-generated separation of carriers in the hybrid materials [24,32]. The efficient electron transfer from PANI to MWCNTs suppressed charge recombination and promoted the generation of reactive oxygen species (e.g., •OH and •O_2_^−^) responsible for methylene blue degradation [37,67]. In addition, the adsorption processes included electrostatic and π–π interactions and the hydrogen-bonding effect on the adsorption of dyes onto the photocatalyst’s surface. The electrostatic attraction was between the cationic dye and negatively charged composite material. Also, photocatalytic ability is highly influenced by the surface chemistry and functionalization of MWCNTs for effective charge carrier separation [68].

**Figure 14:**
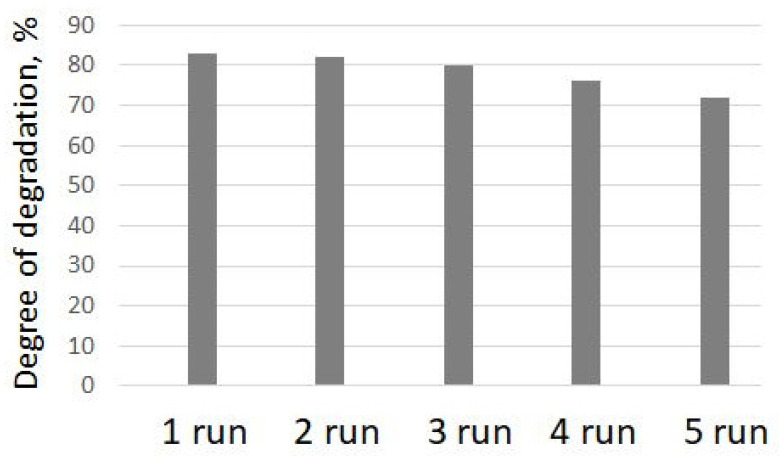
Reusability tests for the c-PANI_H2O2_/CNT-13 hybrid photocatalyst, repeated 5 times, presenting the degree of methylene blue dye degradation after 180 min under UV illumination.

The reusability tests performed with the prepared c-PANI_H2O2_/CNT-13 photocatalyst demonstrated the highest catalytic ability for MB degradation. The studied photocatalyst maintained its photocatalytic activity relatively high after five photocatalytic runs, which confirmed its sustainability (Figure 14).

**Figure 15:**
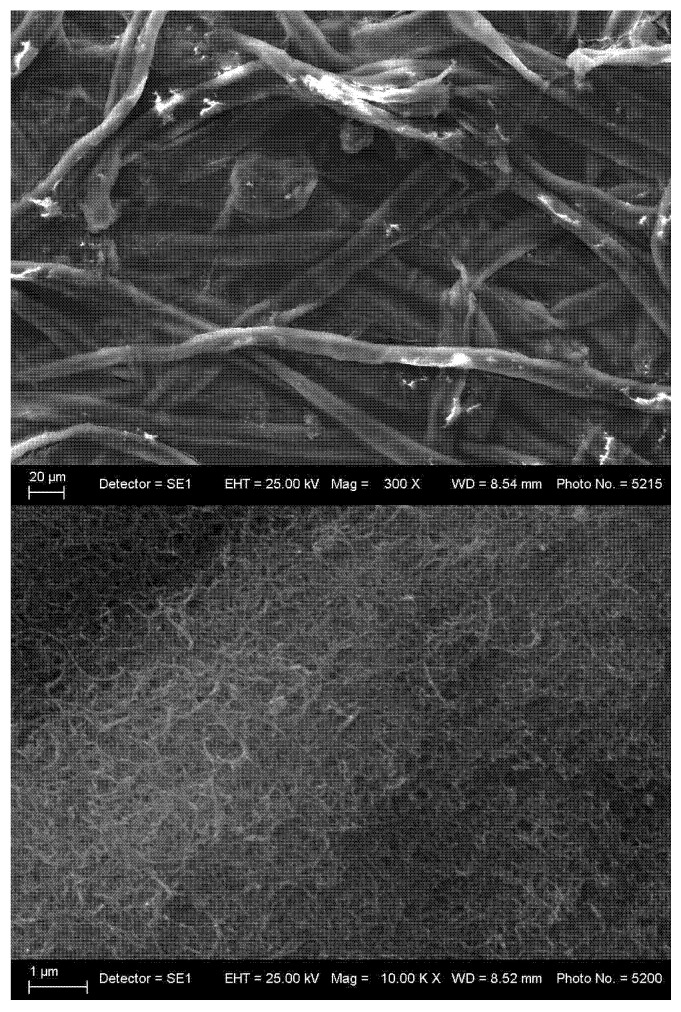
SEM images of c-PANI_H2O2_/CNT-13 spent catalyst after five consecutive photocatalytic runs.

SEM investigations of the spent photocatalyst c-PANI_H2O2_/CNT-13 after photocatalytic degradation of methylene blue dye were performed in order to evaluate the structural stability of the material (Figure 15). The retention of morphology and structure of the spent photocatalyst after the photocatalytic tests was determined by an SEM study. This proved the structural stability of the investigated composite photocatalyst.

## 4. Conclusions

A series of novel hybrid materials based on c-PANI@MWCNTs and cellulose substrates were developed by a facile method involving in situ polymerization of aniline in FA in the presence of MWCNTs and subsequent impregnation of cellulose substrates. Wrapping MWNTs with c-PANI is considered a key factor in achieving stable dispersion in polar solvents such as DMSO and FA and smooth and even deposition onto cellulose substrates. The fabricated c-PANI@MWCNT/cellulose composites demonstrated enhanced photocatalytic ability for degradation of methylene blue dye under UV light compared with the pure c-PANI. The highest degree of degradation of the methylene blue dye (83%) was achieved using the c-PANI_H2O2_/CNT-13 hybrid photocatalyst. The enhanced photocatalytic performance of the c-PANI/MWCNT hybrid composites can be attributed to the synergistic effect of the conductive PANI matrix and carbon nanotubes, which facilitate charge transport and improve interfacial electron charge transfer. In addition, the structural disorder and defects in the MWCNTs may provide additional active sites and contribute to improved photocatalytic efficiency.

## Figures and Tables

**Table 1:** Surface chemical compositions of the obtained cellulose-impregnated samples, as determined by EDS measurement.

Element, Atomic %	cPANI_KPS_-0	cPANI_KPS_/CNT-13	cPANI_KPS_/CNT-26	cPANI_H2O2_/CNT-13	Element, Wt %	cPANI_KPS_-0	cPAN_KPS_/CNT-13	cPANI_KPS_/CNT-26	cPANI_H2O2_/CNT-13
C	86.29	74.81	75.20	65.93	C	81.69	68.68	68.97	59.23
O	12.93	24.86	24.18	34.07	O	16.30	30.28	29.54	40.77
S	0.73	0.42	0.30	-	S	1.84	1.04	0.74	-
K	0.06	-	0.08	-	K	0.17	-	0.23	-
Al	-	-	0.14	-	Al	-	-	0.28	-
Si	-	-	0.11	-	Si	-	-	0.23	-
Total	100.00	100.00	100.00	100.00	Total	100.00	100.00	100.00	100.00

**Table 2:** XPS results of quantitative composition of PANI.

Sample	C, at.%	O, at.%	N, at.%	S, at.%
Cellulose	57.3	42.3	0.4	-
cPANI_H2O2_-0	63.6	34.8	1.6	-
cPANI_H2O2_/CNT-13	79.3	14.1	6.1	0.5
cPANI_KPS_-0	74.2	17.0	7.1	1.7
cPANI_KPS_/CNT-13	69.3	16.8	11.2	2.7
cPANI_KPS_/CNT-26	79.8	12.1	6.2	1.9

**Table 3:** The percentages of C, O and N from various species.

Binding Energy, eV	Chemical Bonding	Concentration, %					
	C1s	Cellulose	c-PANI_H2O2_-0	cPANI_H2O2_/CNT-13	c-PANI_KPS_-0	c-PANI_KPS_/CNT-13	c-PANI_KPS_/ CNT-26
285.0	C-C	2.4	12.5	28.0	19.5	13.4	24.2
286.3	C-O/C-N	29.2	23.8	11.7	15.6	7.0	8.5
288.0	C=O/C=N	27.6	16.9	18.3	23.3	5.8	8.0
289.0	COOH	-	-	21.7	37.0	14.4	26.9
O1s							
531.5	C=O	0.6	21.6	17.0	24.5	17.7	18.6
532.7	C-O	3.6	60.2	11.0	9.1	8.3	7.8
534.1	H_2_O	0.5	8.2	29.5	37.8	10.9	13.1
N1s							
398.6	Imine, -N=C	36.9	2.6	20.3	17.9	10.5	11.8
399.9	Amine, -NH-	-	6.0	22.8	26.5	24.1	20.6
401.3	C-N	17.8	4.6	16.3	24.3	21.2	15.8

**Table 4:** The degree of degradation at 180 min of methylene blue dye.

Sample	Degree of Degradation (%)
c-PANI_H2O2_/CNT-13	83
c-PANI_KPS_/CNT-26	70
c-PANI_KPS_/CNT-13	55
c-PANI_H2O2_-0	42
c-PANI_KPS_-0	27

## Data Availability

The original contributions presented in this study are included in the article/Supplementary Materials. Further inquiries can be directed to the corresponding authors.

## Supplementary Materials

The following supporting information can be downloaded at https://www.mdpi.com/article/10.3390/nano16120735/s1. Video S1: Recorded video of observed free Brownian motion of 0.5 mg·mL^−1^ c-PANI_H2O2_/CNT 13 dispersion in DMSO at digital camera magnification ×20. Video S2: Recorded video of observed free Brownian motion of 0.5 mg·mL^−1^ c-PANI_H2O2_/CNT 13 dispersion in DMSO at digital camera magnification ×100.
